# Arthius on Static Electricity

**Published:** 1874-05

**Authors:** 


TREATMENT OF NERVOUS AND RHEUMATIC AFFECTIONS BY STATIC ELECTRICITY. By Dr. A. Arthius. Translated from the French, by J. H. Etheridge, M.D., Professor of General Therapeutics, Rush Medical College, Chicago. Chicago: W. B. Keen, Cook & Co. 1874. 12 mo. Cloth, pp. 144. Price, $2.00.

"The following translation was made from a desire to contribute to the literature of a subject scarcely known to young American physicians. The use of Static Electricity, in a primitive form, in treating diseases, was well known to medical men in this country, fifty years ago. The application of it, as recommended by the author, is wholly new. The translator is indebted to a friend for a copy of the original, she having received it from a relative in Paris, who was cured of an obstinate nervous disorder by Dr. Arthius' new method."

